# Characterization of Growth Morphology and Pathology, and Draft Genome Sequencing of *Botrytis fabae*, the Causal Organism of Chocolate Spot of Faba Bean (*Vicia faba* L.)

**DOI:** 10.3389/fmicb.2020.00217

**Published:** 2020-02-18

**Authors:** Robert C. Lee, Lina M. Farfan-Caceres, Johannes W. Debler, Robert A. Syme

**Affiliations:** Centre for Crop and Disease Management, School of Molecular and Life Sciences, Curtin University, Bentley, WA, Australia

**Keywords:** necrotroph, phytopathogen, plant pathogen, ascomycete, chocolate spot, gray mold, BGM, lentil

## Abstract

Chocolate spot is a major fungal disease of faba bean caused by the ascomycete fungus, *Botrytis fabae*. *B. fabae* is also implicated in botrytis gray mold disease in lentils, along with *B. cinerea*. Here we have isolated and characterized two *B. fabae* isolates from chocolate spot lesions on faba bean leaves. In plant disease assays on faba bean and lentil, *B. fabae* was more aggressive than *B. cinerea* and we observed variation in susceptibility among a small set of cultivars for both plant hosts. Using light microscopy, we observed a spreading, generalized necrosis response in faba bean toward *B. fabae*. In contrast, the plant response to *B. cinerea* was localized to epidermal cells underlying germinated spores and appressoria. In addition to the species characterization of *B. fabae*, we produced genome assemblies for both *B. fabae* isolates using Illumina sequencing. Genome sequencing coverage and assembly size for *B. fabae* isolates, were 27x and 45x, and 43.2 and 44.5 Mb, respectively. Following genome assembly and annotation, carbohydrate-active enzyme (CAZymes) and effector genes were predicted. There were no major differences in the numbers of each of the major classes of CAZymes. We predicted 29 effector genes for *B. fabae*, and using the same selection criteria for *B. cinerea*, we predicted 34 putative effector genes. For five of the predicted effector genes, the pairwise dN/dS ratio between orthologs from *B. fabae* and *B. cinerea* was greater than 1.0, suggesting positive selection and the potential evolution of molecular mechanisms for host specificity in *B. fabae*. Furthermore, a homology search of secondary metabolite clusters revealed the absence of the *B. cinerea* phytotoxin botrydial and several other uncharacterized secondary metabolite biosynthesis genes from *B. fabae*. Although there were no obvious differences in the number or proportional representation of different transposable element classes, the overall proportion of AT-rich DNA sequence in *B. fabae* was double that of *B. cinerea*.

## Introduction

Faba bean (*Vicia faba* L.) is a widely grown legume crop globally and the three highest-producing countries are China, Ethiopia and Australia (FAOSTAT, 2019^1^). Average production for these top three countries over the recent 5-year period has been 1.6 million tons (MT), 0.9 MT and 0.36 MT, respectively. While Australia is a major faba bean exporter, faba bean production in China and Ethiopia is centered around domestic consumption and subsistence agriculture (Sahile et al., 2008a, b; Li et al., 2017; Yitayih and Azmeraw, 2018). Grains from legume crops are an important source of dietary protein in many countries, particularly in the Middle-East and Northern Africa. For large scale farming systems in Australia, faba beans are an attractive export crop due largely to the contribution they make to nitrogen fixation and soil biodiversity in crop rotations.

Faba bean production is impacted by fungal diseases caused by pathogen species such as *Ascochyta fabae*, *Cercospora zonata*, and *Botrytis fabae* (Tivoli et al., 2006; Stoddard et al., 2010; Kimber et al., 2016). *B. fabae* causes the destructive plant disease commonly called chocolate spot and this disease is distinct from other foliar diseases of faba beans (Harrison, 1988; Tivoli et al., 2006). Epidemics of chocolate spot are initiated in faba bean crops where inoculum is present on crop residues from previous years or from contaminated seed (Harrison, 1978, 1979). *B. fabae* produces conidia from germinated sclerotia that reside in infected stems and are often found in the lodged stubble of the previous year’s crop (Harrison, 1979). Conditions of high relative humidity and rainfall favor saprophytic mycelial growth and germination of sclerotia, and the formation of conidiophores that bear wind and rain splash-dispersed conidia. Favorable environmental conditions such as high crop canopy humidity, consecutive days of rainfall, prolonged leaf wetness and wind combine to promote sporulation, dispersal and germination of conidia on leaf and stem surfaces (Fitt et al., 1985; Harrison, 1988). Intermittent cycles of such favorable conditions lead to epidemics of disease and can cause considerable economic loss through reduced grain yield and poor grain quality (Murray and Brennan, 2012). The severity of chocolate spot epidemics in faba bean production can be mitigated through integrated disease management strategies such as use of clean seed, crop rotation, reduced planting density and selection of more resistant varieties (Stoddard et al., 2010). The timely application of fungicides with demonstrated efficacy against chocolate spot is also an important disease control practice (Sahile et al., 2008b; Stoddard et al., 2010). Only a few sources of genetic resistance to chocolate spot have been identified in faba bean germplasm (Hanounik and Robertson, 1988; Bouhassan et al., 2004; Villegas-Fernández et al., 2009; Maalouf et al., 2016). However, resistant varieties that are adapted to a range of growing regions have not yet been made widely available to farmers (Sillero et al., 2010). The most recent variety releases available to Australian farmers are rated as only having intermediate levels of resistance to chocolate spot, as moderately susceptible (MS) (PBA Samira Variety Guide, Pulse Breeding Australia). Chocolate spot is the most important disease of faba bean in Australia and average annual crop losses and control costs are A$1.4 M and A$5.4 M, respectively (Murray and Brennan, 2012). Disease control measures such as fungicide application and improved varieties help to mitigate the estimated A$8.8 M potential loss due to chocolate spot if disease control strategies were not implemented.

Botrytis gray mold (BGM) of lentil is considered to be a serious plant disease and has commonly been attributed to *Botrytis cinerea* (Bayaa and Erskine, 1998). Research has shown that BGM-infected lentil crops and crop residues contain both *B. fabae* and *B. cinerea* conidia based on species identification by conidial size (Lindbeck et al., 2008, 2009). In addition, both *B. fabae* and *B. cinerea* can infect lentil with similar levels of aggressiveness and there is variability among *B. fabae* isolates in aggressiveness (Davidson and Krysinska-Kaczmarek, 2007). There is consequently an increased risk of BGM in lentil crops in areas where faba beans are grown and chocolate spot disease epidemics are common. It is unclear whether *B. fabae* is a significant contributor to the development and impact of BGM in lentil crops or whether it is simply an opportunistic pathogen that colonizes lentil in conjunction with *B. cinerea*. Further investigation is required to assess the relative proportions of the two *Botrytis* species in BGM epidemics, the capacity for each of the respective species to cause disease in lentil, and whether there are molecular mechanisms that can explain why chocolate spot in faba bean is a distinct disease caused solely by *B. fabae*.

*Botrytis* spp. are ascomycete fungi, classified within class: *leotiomycetes*, family: *sclerotiniaceae*, which includes the major non-host specific plant pathogens *B. cinerea* and *Sclerotinia sclerotiorum* (Bolton et al., 2006; Amselem et al., 2011; Andrew et al., 2012). *B. cinerea* has been characterized as a necrotroph that kills host cells by activating programed cell death and the hypersensitive response (Veloso and van Kan, 2018). In contrast, *S. sclerotiorum* is a hemibiotroph that has a period of biotrophic or asymptomatic growth, followed by a necrotrophic phase (Kabbage et al., 2015). These species have been widely studied and high quality genome sequences have been recently published (Amselem et al., 2011; Derbyshire et al., 2017; van Kan et al., 2017). Genome sequencing of a further nine *Botrytis* species, including several that specifically infect monocot plant hosts, has enabled the identification of candidate virulence genes that are predicted to confer host specificity (Valero-Jiménez et al., 2019). Genes that encode effector proteins or genes from biosynthetic pathways for secondary metabolite effectors are the most important genes that determine host species or cultivar specificity of microbial pathogens. Furthermore, a recent paper describes *Botrytis fabiopsis* that causes chocolate spot disease in faba beans in China and is closely related but distinct from *B. fabae* (Zhang et al., 2010). There are a limited number of *B. fabae* gene sequences in the GenBank database and these have enabled the phylogenetic classification of *B. fabae* with other *Botrytis* and *Sclerotinia* species (Zhang et al., 2010).

Genome sequencing has driven major advances in the study of mechanisms of pathogenicity and virulence in fungal species that cause plant disease. This has been particularly the case for the identification and characterization of effectors and avirulence proteins that mediate the interactions of microbes with plants in order to achieve pathogenic lifestyles. An example of the comparative genomics approach to studying related species is the recent comparison of the genomes of 18 dothideomycete species, most of which are plant pathogens with diverse lifestyles (Ohm et al., 2012). This study assessed the complement of carbohydrate and protein-degrading enzymes, and small secreted proteins among other protein classes to make inferences about genome and gene evolution, and ecological and pathogenic specialization (Ohm et al., 2012). The suite of effectors produced by *B. cinerea* and *S. sclerotiorum* are not well characterized although genome sequencing has allowed effector and virulence gene candidates to be predicted (Amselem et al., 2011; Derbyshire et al., 2017; Valero-Jiménez et al., 2019). Protein effector candidates are predicted on the basis of small size and a high proportion of cysteine residues. The presence of a predicted signal peptide for secretion to the apoplast and a lack of homologous sequences among other species are further key signatures for necrotrophic effectors of apoplastic plant pathogenic fungi. Computational approaches have been developed to predict effectors from genomic sequences (Sonah et al., 2016; Jones et al., 2018) and EffectorP is a useful program that rates the likelihood of a protein sequence encoding an effector using a machine-learning approach with validated effectors as training data (Sperschneider et al., 2016, 2018). In addition, secondary metabolites botcinic acid and botrydial are known phytotoxic compounds with established roles in plant disease for other *Botrytis* species and it is not known whether these compounds contribute to plant diseases caused by *B. fabae* (Collado and Viaud, 2016).

In this study, our aim was to classify and characterize two *B. fabae* isolates that were isolated from faba bean crops in Western Australia, and to produce draft genome assemblies from Illumina sequencing data. We have fully characterized the colony morphology of *B. fabae* grown on solid growth media and the features of conidiophores and conidia and compared these to those of *B. cinerea*. In pathology assays, we have assessed the ability of the isolates to infect faba bean and lentil, and we used microscopy and histology to observe the progress of disease in host plants. The two published genome assemblies for *B. fabae* will provide essential information for the future study of the species toward a more complete understanding of chocolate spot disease in faba bean and BGM in legumes such as lentil.

## Materials and Methods

### Isolate Collection and Culture Conditions

Two *B. fabae* isolates were cultured from chocolate spot lesions collected from infected faba bean plants of the variety PBA Samira, in farmers’ crops near Esperance in Western Australia in 2016. Single spore isolates were cultured by surface sterilization (1 min immersion in 1% sodium hypochlorite solution, 1 min 5% ethanol and 1 min sterile water) and grown on 1/2 strength potato dextrose agar (1/2 PDA). Plates were maintained at room temp (18–20°C) under long wave UV light with a 12 h photoperiod. Two single spore isolates were produced from the two samples and these were designated SCD-16-611 and DLY-16-612, abbreviated in this paper as *Bf*611 and *Bf*612, respectively. *B. cinerea* isolate Bc7 was obtained from Dr. Francisco Lopez-Ruiz (Centre for Crop and Disease Management, Curtin University, Australia) for inclusion in growth morphology and pathology studies (Harper et al., 2019). *B. fabae Bf*611, *Bf*612 and *B. cinerea* Bc7 were maintained as agar plugs at 4°C for routine use in the laboratory.

### Characterization of Fungal Growth and Sporulation

Radial growth rates for two *B. fabae* isolates and the *B. cinerea* control isolate were determined by inoculation of a 5 mm diameter section of agar colonized with mycelium from 2-day-old cultures onto the center of a 1/2 PDA plate and grown in the dark at room temperature (20°C). The colony diameter increase 1–2 days after inoculation at the center of the plate was used to measure growth rate (*n* = 5).

The plate morphologies of the two *B. fabae* isolates were compared with the *B. cinerea* control on two types of growth media; 1/2 PDA and 1/2 PDA supplemented with faba bean extract. Half PDA was prepared by adding 19 g potato dextrose agar and 7.5 g agar to 1 L water. Faba bean extract 1/2 PDA was made by substituting the water for an extract of faba bean leaves. To produce the faba bean leaf extract, green and non-senescent faba bean leaves from mature plants (3–4 months age) grown in the field were frozen at −20°C. Thawed, wilted plants were extracted under pressure using a hydraulic press and the extract was filtered through Miracloth and autoclaved. Coagulated material after autoclaving was removed by filtration through Miracloth. Plates were inoculated by placing a 5 mm diameter section of 2 day-old culture from 1/2 PDA at the center of the plate and growth was at room temperature in the dark for 3 days, followed by a further 3–9 days under long wave UV light as above. Plates were photographed at 6 and 12 days after inoculation.

For the production of conidia from *B. fabae*, 1/2 PDA plates were inoculated by spreading a lawn of crushed plate-grown mycelium across 90 mm round or 120 mm square plastic Petri plates. The plates were grown in the dark at room temperature and at 6 days, surface-sterile fresh faba bean seedling leaves from 2–3 weeks old plants, were placed in close contact with the actively growing mycelium on the plate surface. Plates were sealed with plastic wrap and incubated at room temperature under long wave UV light with a 12 h photoperiod as above. After a further 6 days leaves were removed from the plates, placed in 50 mL plastic conical centrifuge tubes (Falcon type) and covered with approx. 40 mL sterile water. The tubes were shaken vigorously by hand and the spore suspension filtered through open weave muslin cloth in a 50 mL plastic syringe. Spores were counted using a hemocytometer on a benchtop light microscope.

### Pathology Studies and Microscopy

We observed and photographed conidiophores produced on plated infected faba bean leaves using an Olympus SZH10 (Olympus, Tokyo, Japan) dissecting microscope with overhead illumination image capture using an Olympus digital camera connected to Olympus Master 2.06 software. For microscopic imaging of conidiophores and conidia, we used an Olympus BX51 light microscope (Olympus, Tokyo, Japan) with image recording using the Olympus DP controller image capture software. For mounting intact conidiophores, we used clear adhesive tape and lightly contacted the fungal growth from faba bean leaves on plates and placed the tape with adhered fungal material face down on a droplet of water on a glass microscope slide. Observations were made using bright field settings and 10x and 20x objectives.

We conducted three plant infection experiments on 2–3 week-old seedlings inoculated with 4.0 × 10^4^ spores.mL^–1^ in water with 0.05% v/v Tween 20 (Sigma-Aldrich, St. Louis, MO, United States). Plants were grown in potting soil (UWA mix; Richgro, Jandakot, Australia) in 5 cm square plastic pots with approx. 7 cm depth. Two to three seeds were sown for each of six replicate pots (*n* = 6) and plants were grown in a controlled environment growth facility at 18–22°C under 5000 K cool-white LED lights (Screen-Tech, Canning Vale, Australia). Experimental treatments of cultivar and isolate were tested using a split-block design. Disease severity (DS) was scored as an approximation by visual assessment, of the percentage of the leaf area affected by necrosis, at four days after infection. For experiment 3 where disease progression was slow, disease assessments were made also at seven days after infection.

For the examination of fungal colonization of infected faba bean and lentil leaves from plant infection experiments, we sampled leaves with necrotic lesions at defined time points after inoculation. Leaves were placed in clearing solution (ethanol:glacial acetic acid; 1:2 v/v) for 24 h, and for a second 24 h period in fresh clearing solution followed by staining in 0.05% w/v Trypan Blue (Sigma-Aldrich) in 50 mM phosphate buffer, pH 7.0. Observation and image collection was as described above using the Olympus BX51 light microscope and Olympus image capture software.

### Statistical Analysis

Plate growth rates and pathology assay data were processed using One-Way ANOVA and Tukey-HSD multiple pairwise comparisons using the R statistical computing packages dplyr and ggpubr (downloaded from https://cran.r-project.org) in RStudio.

### DNA Isolation and Genome Sequencing

Fungal cultures were grown in yeast extract glucose (2-YEG) (yeast extract 2 g.L^–1^ and glucose 10 g.L^–1^) in an 80–100 mL volume in 250 mL Erlenmeyer flasks, with shaking at 180 rpm for 3 days at 22°C. Mycelium was collected, ground under liquid nitrogen and freeze-dried overnight (ScanVac Coolsafe 55-4 freeze dryer; LaboGene, Sweden). Genomic DNA from *B. fabae* isolates *Bf*611 and *Bf*612 was extracted from fungal mycelia using the modified method of Xin and Chen (Xin and Chen, 2012). DNA samples were dissolved in 100 μL 10 mM Tris-HCl (pH 7.0) and purified by 30 min incubation with 180 μL Agencourt AMPure XP magnetic beads (Beckman-Coulter, Indianapolis, IN, United States). The beads with bound DNA were washed twice with 200 μL 80% v/v ethanol. Clean DNA was eluted with 55 μL 10 mM Tris-HCl pH 8.0. DNA concentrations were determined using a Qubit 2.0^®^ fluorometer (Invitrogen, Carlsbad, CA, United States) with the dsDNA broad range assay kit (Thermo-Fisher, Waltham, MA, United States) and NanoDrop^®^ Spectrophotometer (Thermo-Fisher). DNA quality and purity were assessed by agarose gel electrophoresis.

Whole genome sequencing of *B. fabae Bf*611 and *Bf*612 was undertaken by the production of Nextera (Illumina, San Diego, CA, United States) genomic libraries and sequencing on the Illumina NextSeq platform. Sequencing libraries were prepared with 50 ng of genomic DNA for each isolate using the Nextera DNA library preparation kit and the compatible Nextera index kit (Illumina) following the enclosed product reference guides. Multiplexed libraries were loaded onto the Illumina NextSeq 500 instrument and 150 bp Paired-End reads were acquired and saved to the BaseSpace archive (Illumina).

### Genome Assembly and Analysis

Genome sequencing quality for raw Illumina data was assessed using FastQC 0.11.5^2^ and indexed reads were trimmed using Trimmomatic 0.38 (Bolger et al., 2014). Contigs were assembled using SPAdes 3.11.1 (-k 21,33,55,77,99,127 –careful) (Bankevich et al., 2012). Sequencing statistics were calculated from the draft assemblies for each of the two isolates using Quast 4.6.2 (Gurevich et al., 2013).

Initial gene prediction for *Bf*611 and *Bf*612 was performed using GeneMark-ES 4.33 (options: –ES –fungus) (Lomsadze et al., 2005; Ter-Hovhannisyan et al., 2008). tRNAscan-SE 1.3.1 (Lowe and Eddy, 1997) was used for the prediction of tRNA genes and Infernal 1.1.2 (Nawrocki and Eddy, 2013) was used for identification of non-coding RNA sequences, including tRNA genes, ribosomal RNA and long non-coding RNA sequences. Repetitive DNA sequences were predicted using RepeatModeler Version 1.0.11^3^ to construct a repeat library from the published *B. cinerea* genome (B05.10 ASM14353v4, GenBank assembly accession GCA_000143535.4) and then running RepeatMasker using the *B. cinerea* library on the *Bf*611 and *Bf*612 genome assemblies (RepeatMasker Version open 4.0.7^2^). For final genome annotation and gene model prediction we downloaded all RNASeq datasets labeled ‘*Botrytis cinerea*’ (see Supplementary Data for SRA reference numbers) and used exonerate (Version 2.2.0) (options -E false –model p2g –percent 80 –geneseed 250)^4^ to create CDS hints for Augustus (Version 3.3) (Stanke et al., 2004, 2006; König et al., 2016). For the assessment of genome completeness and genome assembly quality, we used BUSCO (Version 3.0.2) (Simão et al., 2015; Waterhouse et al., 2017) and comparative assessments were made using the “Ascomycota” fungal data sets downloaded from the BUSCO website https://busco.ezlab.org/. GC content was calculated using the OcculterCut software package Version 1.1, https://sourceforge.net/projects/occultercut/(Testa et al., 2016).

We prepared graphical representations of the two draft *B. fabae* genomes using NUCmer (Version 3.1) (Delcher et al., 1999; Kurtz et al., 2004), with alignment of the *B. fabae* contigs to the reference *B. cinerea* B05.10 chromosomes (van Kan et al., 2017). “Unique” and “non-unique” DNA sequence matches were highlighted to indicate regions of likely repetitive and non-coding DNA sequence in regions of “non-unique” matches.

### Analysis of Transposable Elements (TE) and Repetitive DNA

We used the PiRATE-Galaxy pipeline virtual machine using VMware Workstation 15 Player version 15.0.4^5^ to run transposable element detection, clustering and classification steps as recently described (Berthelier et al., 2018). Input data files were FASTA contigs files for *B. fabae* and the 18 chromosomal contigs from the reference *B. cinerea* B05.10 assembly (van Kan et al., 2017). In PiRATE-Galaxy, bioinformatics software and file management systems are packaged together in a GUI-based, stand-alone Galaxy server instance in the virtual machine (Giardine et al., 2005). Detection of repetitive and transposable element sequences was completed using similarity-based detection programs RepeatMasker^6^ and TE-HMMER (Berthelier et al., 2018), a custom program based on HMMER (Eddy, 1996) and tBLASTn (Altschul et al., 1990); structure-based programs MITE-Hunter (Han and Wessler, 2010), SINE-Finder (Wenke et al., 2011), Helsearch (Yang and Bennetzen, 2009) and LTRharvest (Ellinghaus et al., 2008); and repetitiveness-based programs, TEdenovo (Flutre et al., 2011) and RepeatScout (Price et al., 2005). Output files from RepeatMasker, TE-HMMER, Helsearch, LTRharvest, TEdenovo from the REPET package, and RepeatMasker, were concatenated into a single FASTA file for each isolate. From these TE and repeat DNA files, short sequences that form part of a larger 100% identical sequence elsewhere in the file were removed using the program CD-HIT-est (Li and Godzik, 2006) in PiRATE-Galaxy to reduce redundancy in the combined TE and repeat sequence dataset. Short sequences of less than 200 nucleotides in length were also removed from further analysis. The classification step was carried out using PASTEC (Hoede et al., 2014) with the nucleotide, protein and profile HMMs databanks implemented in the PiRATE-Galaxy server.

### CAZymes and Effector Predictions

We performed an assessment of the predicted proteome set for the two *B. fabae* isolates by classification of carbohydrate-active enzymes (CAZymes) and putative protein effectors. As a first step we used DeepSig (Version 1.0) https://deepsig.biocomp.unibo.it/deepsig and SignalP (Version 4.1) (Petersen et al., 2011) to predict the set of secreted proteins. The dbCAN2 webserver (Zhang et al., 2018) was used for the prediction and classification of CAZymes. As suggested by dbCAN2 we annotated a CAZyme if it was predicted by at least two of the three programs run by the dbCAN2 webserver. dbCAN2 runs HMMER against the dbCAN HMM database, DIAMOND (Buchfink et al., 2015) against the CAZy pre-annotated CAZyme database and Hotpep (Busk et al., 2017) against the conserved CAZyme short peptide database. We selected putative effector proteins from the set of secreted proteins predicted by both DeepSig and SignalP 4.1, with preference for SignalP when there was disagreement on the signal peptide cleavage site. Further selection was on the basis of mature protein size less than 25 kDa, EffectorP score greater than or equal to 0.8 (Version 2.0) (Sperschneider et al., 2018) and at least two cysteines. We applied the same CAZymes and effector prediction methods to the protein set for the published *B. cinerea* B05.10 genome assembly from the NCBI archive (van Kan et al., 2017). We used dNdS-Calculator^7^ (Shaw et al., 2012) to calculate pairwise dN/dS ratios for putative effectors in *B. fabae* with orthologous genes in *B. cinerea*.

### Other Protein Families and Ortholog Groups

To classify protein families other than effectors and carbohydrate-active enzymes, we used InterProScan^8^ (version 5.29-68.0)^9^ to assign gene ontology descriptors to annotated proteins and OrthoFinder (Version 2.2.3) https://github.com/davidemms/OrthoFinder (Emms and Kelly, 2015) to assign predicted proteins to ortholog groups across the two *B. fabae* isolates and *B. cinerea*. A Venn diagram output from OrthoFinder was produced to compare orthologous gene groups shared by all three genome assemblies and between pairs of assemblies used in the study. Secondary metabolite clusters were identified using BLASTp and tBLASTn (Altschul et al., 1990) and the antiSMASH v 5.0 fungal webserver (Medema et al., 2011; Blin et al., 2019).

### Data Availability

Draft genome assemblies for *Botrytis fabae* SCD-16-611 (referred to herein as *Bf*611) and DLY-16-612 (*Bf*612) are available under the NCBI BioProject PRJNA505227. SRA data is available at NCBI and the WGS accession numbers are GCA_004335035.1 (SCD-16-611) and GCA_004335055.1 (DLY-16-612). All other data is available within the article and Supplementary Files.

## Results

### Characterization of *Botrytis fabae* Isolates

We sampled chocolate spot-infected faba bean leaves from established commercial crops in the Scaddan and Dalyup area near Esperance in Western Australia in the early spring season of 2016. Chocolate spot disease symptoms were widespread in the lower canopy of the crop, owing to the high level of rainfall for that season. In 2016, from June to September, over 300 mm rain fell in the Scaddan area with the average rainfall for the same period in previous years being 225 mm (Australian Government Bureau of Meteorology data).^10^ Chocolate spot lesions were identified by red-brown areas of necrosis on foliage and other diseases of faba beans such as ascochyta blight were not evident. We cultured two single spore isolates and named these for the approximate locations of the farms where the samples were taken, SCD-16-611 (*Bf*611) and DLY-16-612 (*Bf*612).

**FIGURE 1:**
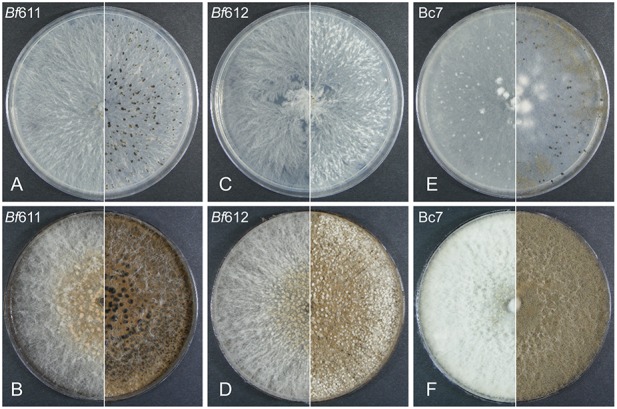
Plate morphology for *B. fabae Bf*611 **(A,B)**, *B. fabae Bf*612 **(C,D)**, and *Botrytis cinerea* Bc7 **(E,F)**, on 1/2 PDA (top row **A**,**C**,**E**) or faba bean extract 1/2 PDA media (bottom row **B**,**D**,**F**). Plates were inoculated with a 5 mm diameter plug from a 2-day-old plate for each isolate and maintained in darkness for 6 days (left panel) and 12 days (right panel). Images are representative of five replicate plates for each growth media and isolate combination.

Growth rates on 1/2 PDA plates were measured for the two *B. fabae* isolates and compared to that for *B. cinerea*. We found that growth rates for *Bf*611 and *Bf* 612 were not significantly different to each other (*p* < 0.5) but were significantly different to Bc7 (*p* < 0.05). Average diameter increase for growth on 1/2 PDA from 24 h to 48 h was 20 mm for the *B. fabae* isolates and 28 mm for Bc7. For the visual differentiation of the putative *B. fabae* isolates on solid plate media, we used 1/2 PDA and faba bean extract-supplemented 1/2 PDA to look for differences in morphological features with *B. cinerea* that could be used for differentiation of the two species. We assumed that *B. cinerea* would possibly be present in our collected samples and in field samples from other legumes that may exhibit BGM symptoms. Visual differentiation of the two species would be useful for future sampling and diagnostic studies for diseases in legume crops caused by *Botrytis* species. We used the *B. cinerea* isolate Bc7 for comparisons with *B. fabae* isolates *Bf*611 and *Bf*612 (Figure 1). On 1/2 PDA at 6 days post inoculation (DPI) morphological characteristics were similar with only a more striated pattern of hyphal growth for the two *B. fabae* isolates to differentiate these from *B. cinerea*. At 12 DPI the hyphal striations were still a minor differentiating feature for *B. fabae*, and sclerotia were beginning to appear for both species. The formation of sclerotia for *Bf*612 was delayed in comparison to *Bf*611, and *B. cinerea* Bc7 also produced sclerotia at an intermediate level. Brown conidia were evident on *B. cinerea* plates at 12 DPI but appeared to be completely absent for the two *B. fabae* isolates. We held the view that the addition of host plant compounds in growth media in the form of an extract from faba bean leaves might induce altered growth morphology in each of the isolates by exposure to additional plant-derived nutrients or secondary metabolites. Figure 1 shows greatly increased mycelial growth for *B. cinerea* at 6 DPI with the addition of plant extract to the culture media and at 12 DPI there were masses of gray-brown conidia. In contrast, conidia were absent for the two *B. fabae* isolates at 12 DPI on the faba bean extract-supplemented media. For the two *B. fabae* isolates, initiation of sclerotia formation was also observed at the 6 DPI time point. Similar to growth on non-supplemented 1/2 PDA, the sclerotia of *Bf*612 were less well developed and not as dark colored as those for *Bf*611. Overall, the most distinguishing morphological feature for the *Botrytis* species on plates was the profuse production of conidia for *B. cinerea* and lack of conidiation for *B. fabae*, and this was more pronounced on growth media to which faba bean extract had been added.

**FIGURE 2:**
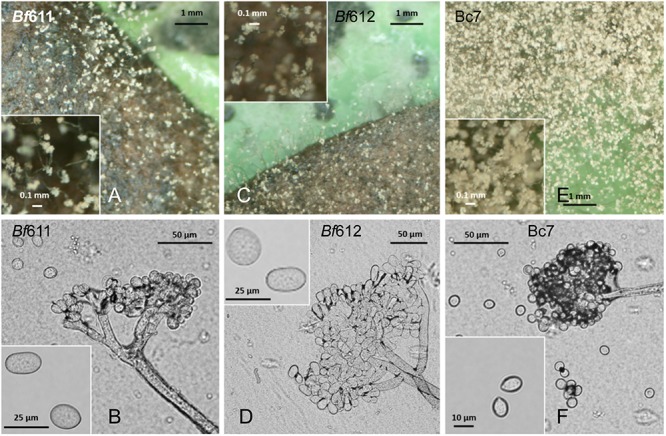
Spore production on 1/2 PDA plates with faba bean leaves (top row **A**,**C**,**E**) and conidiophores and conidia (bottom row **B**,**D**,**F**) for *B. fabae Bf*611 **(A,B)**, *Bf*612 **(C,D),** and for *B. cinerea* Bc7 **(E,F)**. Plates were inoculated with a mycelial suspension from a pre-grown plate culture and grown in the dark for 3 days. Surface-sterilized leaves were overlaid on the growing mycelium and plates maintained at room temperature for 6 days. Conidiophores and conidia were observed by stereo microscope and bright-field light microscopy at 6 days after placing the faba bean leaves.

The scant production of *B. fabae* conidia was problematic for the proposed pathology assays and we experimented with alternative growth media such as MediaX documented by Last and Hamley (1956; Leach and Moore, 1966), but without reliable success. We adapted a simple method whereby sporulation would be induced by the presence of host leaves on fungal cultures on plates as suggested in Tivoli et al. (2006). As detailed in the section “Materials and Methods,” we overlaid sterilized faba bean leaves onto the mycelial growth on 1/2 PDA plates. The *B. fabae* isolates colonized the leaves and after approximately 6 days conidiophores bearing conidia were observed (Figure 2). Under the same conditions, *B. cinerea* similarly produced conidia and at an observably greater number than *B. fabae*. Although spore numbers were low for *B. fabae*, using this method we were able to produce spores for microscopic observation and plant pathology assays. Light microscopy revealed *B. fabae* as having branched conidiophores of generally larger size and with fewer spores than *B. cinerea*. Conidial size for *Bf*611 and *Bf*612 was approximately 20 μm in length compared with 10 μm for *B. cinerea* Bc7, and these measurements were as has been previously reported (Harrison, 1988; Lindbeck et al., 2008).

### Plant Pathology Assays

**FIGURE 3:**
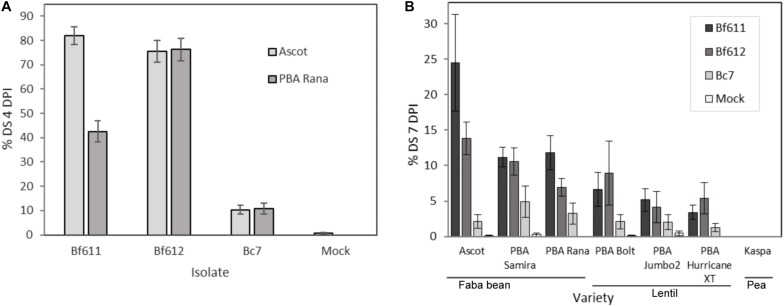
Percent disease severity (% DS) for three experiments measuring disease severity of *B. fabae* and *B. cinerea* infection in faba bean and lentil. **(A)** Experiment 1 and 2: Faba bean cultivars Ascot and PBA Rana at 4 DPI; **(B)** Experiment 3: Faba bean varieties (Ascot, PBA Samira and PBA Rana), lentil varieties (PBA Bolt, PBA Jumbo2 and PBA Hurricane XT) and field pea variety Kaspa, at 7 DPI. Error bars show plus/minus standard error.

We tested the susceptibility of faba bean seedlings to infection by the *B. fabae* isolates *Bf*611 and *Bf*612 and made comparisons to infection with *B. cinerea* isolate Bc7. The results of these assays are presented in Figure 3. In two independent experiments faba bean varieties Ascot and PBA Rana (Figure 3A) were inoculated with 4.0 × 10^4^ spores.mL^–1^ of each of the two *B. fabae* isolates or the *B. cinerea* control. At 4 DPI, *B. fabae* infections were severe with up to 80% necrosis, and large, coalescing lesions compared with 10% leaf area necrosis and smaller isolated lesions for *B. cinerea*. For faba beans, *Botrytis cinerea* Bc7 disease scores were not significantly different to the mock treatment (*p* < 0.1). Faba bean cultivar Ascot was susceptible to *Bf*611 and *Bf*612 to a similar degree (*p* < 0.001), however, PBA Rana displayed reduced susceptibility to *Bf*611 in comparison to *Bf*612 (*p* < 0.001) with only 40% leaf area necrosis. In an assessment of the *B. fabae* susceptibility of lentils and to address the question of a potential role for *B. fabae* in causing lentil BGM we ran disease assays on three lentil varieties alongside three faba bean varieties (Figure 3B). We assessed disease progression at both 4 DPI (not shown) and at 7 DPI (Figure 3B) and applied statistical tests to the 7 DPI scores. Faba bean variety Ascot was more susceptible to disease caused by *B. fabae Bf*611 than both varieties PBA Rana and PBA Samira (*p* < 0.005). The three lentil varieties displayed low levels of disease symptoms to *B. fabae* with *Bf*612 producing significant disease symptoms compared to the mock infection treatment for PBA Hurricane XT (*p* < 0.001), PBA Bolt (*p* < 0.05) and PBA Jumbo2 (*p* < 0.1). Isolate *Bf*611 produced significant levels of disease only on PBA Hurricane XT (*p* < 0.1). Interestingly, *B. cinerea*, often considered to be the main causal organism of BGM in lentil, was consistently less aggressive in terms of necrosis symptoms than *B. fabae* on the three lentil varieties, with disease scores for Bc7 not significantly different to the mock infection on lentils (*p* < 0.5). As a control, field pea variety Kaspa displayed no disease symptoms when inoculated with either *B. fabae* or *B. cinerea*.

**FIGURE 4:**
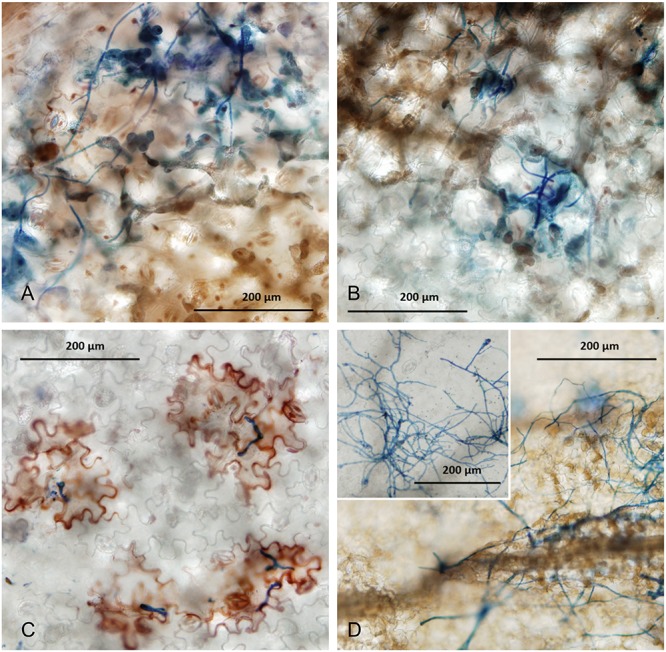
Samples of infected leaves were collected from seedling infection experiments at 4 DPI. Cleared specimens were stained with Trypan Blue stain and observed under a light microscope using bright field settings. **(A,B)** Faba bean variety PBA Rana 6 DPI with *B. fabae Bf*611 **(A)** and *Bf*612 **(B)**. **(C)** Faba bean variety PBA Rana 6 DPI with *B. cinerea* Bc7. **(D)** Lentil varieties PBA Bolt (main image) and PBA Jumbo2 (inset) 6 DPI with *B. fabae Bf*612.

Using light microscopy on cleared and Trypan Blue-stained infected leaves, we observed the colonization and necrosis in faba bean and lentil leaves resulting from plant infection by *Botrytis* species (Figure 4). *B. fabae* infection in faba beans produce spreading necrosis, and hyphal colonization of the surface of infected leaves around the site of macroscopic necrotic lesions (Figures 4A,B). In contrast to the compatible infection response that was evident for *B. fabae* in faba bean, *B. cinerea* on faba bean had less hyphal growth at 6DPI and infection appeared to induce an immune response as observed by red-brown coloration of epidermal cells beneath germinated spores and appressoria (Figure 4C). Necrosis was localized only to epidermal cells that we observed as directly interacting with *B. cinerea* and possibly to one or two neighboring cells. Also noteworthy was the inhibition of hyphal development on the leaf surface. It appeared that *B. cinerea* was unable to traverse the leaf surface to attempt penetration at other locations. Our observations for faba beans are evidence that *B. fabae* and *B. cinerea* are markedly different in their virulence toward faba beans.

Lentil variety PBA Jumbo2 is rated as R (resistant) to BGM and PBA Bolt is rated S (susceptible) (PBA Jumbo2 variety brochure).^11^ In considering the susceptibility of lentils to *B. fabae* in the context of the roles and relative contributions of the two *Botrytis* species in lentil BGM, we observed the reaction of lentil varieties PBA Jumbo2 and PBA Bolt to *B. fabae Bf*612, 6 DPI (Figure 4D). In the more susceptible lentil variety PBA Bolt (Figure 3D), the generalized necrosis was similar to that for the compatible reactions in faba bean and the surface growth of hyphae was extensive. For the more BGM-resistant lentil variety PBA Jumbo2, necrosis was reduced or absent despite the extensive growth of hyphae.

### Genome Sequencing and Assembly

We sequenced the genomic DNA from the two *B. fabae* isolates, *Bf*611 and *Bf*612 using Paired-End sequencing of Nextera libraries on the Illumina NextSeq platform. Sequencing coverage for the Illumina sequencing of *B. fabae* isolates was 27x and 45x for *Bf*611 and *Bf*612 respectively. The assembly of the *Bf*611 and *Bf*612 genomes produced 4,497 contigs in 43.7Mb and 4,152 contigs in 44.5 Mb respectively, with contig N50 and L50 of 24,000 and 500 bp for both assemblies, respectively (Table 1). These assembly sizes are slightly larger than the PacBio *B. cinerea* B05.10 reference assembly that is 43.5 Mbp (Table 1) (van Kan et al., 2017). For both *B. fabae* isolates, four contigs comprised the mitochondrial genome, which totaled 84,436 bp and 75,998 bp for *Bf*611 and *Bf*612 respectively. SRA data for genome sequencing of the two isolates are available on the NCBI database under the BioProject PRJNA505227. Genome assemblies are archived on the NCBI WGS database under accession numbers GCA_004335035.1 and GCA_004335055.1, for *Bf*611 and *Bf*612, respectively.

**TABLE 1:** Genome assembly and annotation statistics for *Botrytis fabae Bf*611 and *Bf*612, compared to the published *B. cinerea* B05.10 reference genome (van Kan et al., 2017).

**Parameter**	**Illumina draft genomes**		**PacBio reference**
	***Bf*611**	***Bf*612**	**B05.10 ^a^**
**Sequencing statistics**			
Total read length (Gb)	1.19	2.01	2.76
Genome assembly size (Mb)^b^	43.7	44.5	43.5
Coverage^b^	27x	45x	35x
Total no. of nuclear contigs^b^	4,497	4,152	18 chromosomes
No. of mitochondrial contigs	4	4	–
Size of mitochondrial genome (bp)	85,436	75,998	–
Largest contig (Kb)	121	130	–
L50	513	529	–
N50	23,974	24,070	–
**Annotation statistics**			
No. of predicted genes	11,258	11,433	11,701
% GC content	41.0	40.6	42.0^c^
No. of CAZymes	588	589	681
Predicted effectors^d^	27–67	27–67	34–89

*^*a*^van Kan et al., 2017; ^*b*^Contigs > 500 bp; ^*c*^QUAST (this study); ^*d*^Prediction pipelines detailed in the section “Materials and Methods” (this study), range depending on selection criteria (see Table 2).*

Annotation and gene prediction for *B. fabae* assemblies were performed using a series of methods including GeneMark-ES, repeat masking and evidence-based gene prediction using Augustus with published *B. cinerea* RNAseq data sets. The numbers of predicted genes for the two *B. fabae* isolates were similar to each other at approximately 11,400 protein-coding genes (Table 1). This number is fewer than the 11,701 *B. cinerea* protein-coding genes, however, the better quality PacBio genome assembly for *B. cinerea* is likely to have a more accurate set of predicted genes.

BUSCO gene and protein assessments of genome assembly completeness (Supplementary Figure S1) compared favorably to the more complete *B. cinerea* B05.10 genome (van Kan et al., 2017). Of the 1,315 genes or proteins in the respective comparisons, 98.2–98.6% of genes were complete and single-copy for *B. fabae* and this value was 99.2% for *B. cinerea*. There were more fragmented genes for *Bf*612 (0.91%) than for *Bf*611 (0.38%), compared with 0.076% fragmented genes for *B. cinerea*. Missing genes for *B. fabae* isolates accounted for 0.91 – 1.1% of the benchmarking gene set compared with 0.76% for *B. cinerea*. BUSCO assessment of annotated proteins from the genome assemblies indicated similar levels of fragmentation and missing genes for the two *B. fabae* isolates. The published *B. cinerea* genome assembly had almost no fragmented proteins (3) and no missing proteins. BUSCO proteins analysis identified 1.3 – 1.7% of proteins missing and fragmented proteins in the annotated proteins of the *B. fabae* assemblies. Worth noting was that a large number of proteins (105) for *B. cinerea* were duplicated in the BUSCO proteins analysis. It is possible that these duplications arise from the splicing variants for 181 genes on the B05.10 Chr1 (van Kan et al., 2017).

For the genome-wide comparisons of annotated proteins between the two *B. fabae* isolates and between *B. fabae* and *B. cinerea*, we used the program OrthoFinder to generate orthologous gene groups for each isolate and for the reference, *B. cinerea*, from published data (van Kan et al., 2017). We subsequently compared the members of each orthogroup for each of the three assemblies using homology-based comparisons. Keeping in mind the inherent limitations of comparisons between PacBio and Illumina assemblies, we nevertheless speculate on the similarities between the *Botrytis* species. The sequencing, assembly and analysis of two independent *B. fabae* genomes improves the reliability of the comparisons we make in this study. Analysis using OrthoFinder (Supplementary Figure S2) found that 10,226 orthologous gene groups are represented in all three genome assemblies and 1,622 are in *B. cinerea* but not in *B. fabae*. There were 481 orthogroups found in *B. fabae* and supported by data for the two *B. fabae* assemblies, which were not in *B. cinerea*. There were orthogroups predicted for one or the other of the two *B. fabae* isolates (127 for *Bf*611; 241 for *Bf*612). However, these are less reliable predictions and we only make inferences about the differences between species based on OrthoFinder results using the more complete PacBio assembly and annotation for *B. cinerea*, and for results corroborated by two independent annotated genome assemblies for *B. fabae*.

**FIGURE 5:**
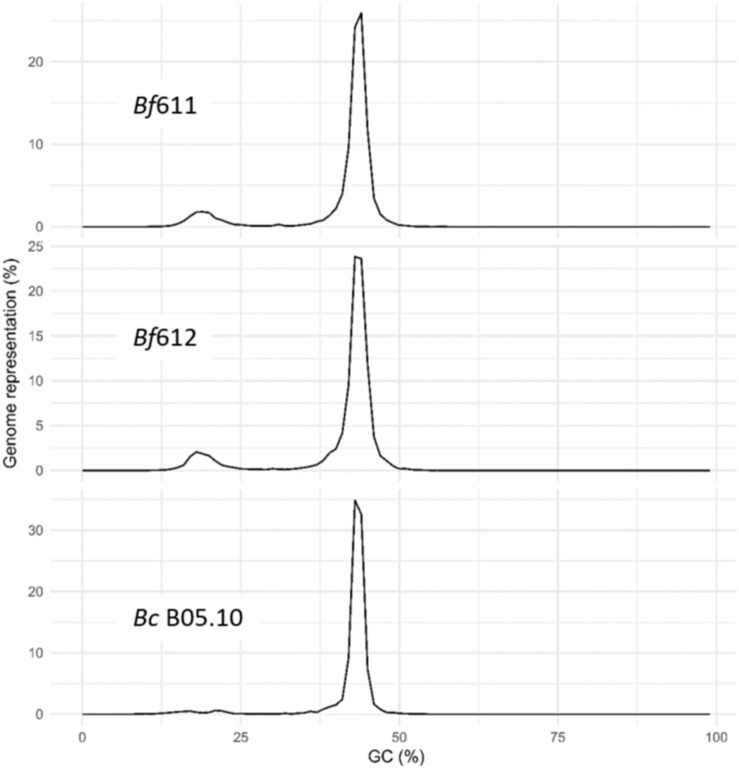
Histograms of percentage genome representation by GC content, for *Botrytis fabae Bf*611 and *Bf*612, and *B. cinerea* B05.10. Analysis was performed using the OcculterCut software package.

We used the OcculterCut package to determine the distribution of percent GC content for the *B. fabae* genomes compared with our OcculterCut analysis of the published *B. cinerea* genome (Figure 5). Analysis of % GC distribution revealed a bi-modal pattern of nucleotide content for *B. fabae* with the main balanced GC content peak at around 43% and a second minor peak of AT-rich DNA at around 18% GC. For both *B. fabae* genomes, the minor AT-rich peak accounted for approximately 10.6–11.3% of the genome assemblies, whereas in *B. cinerea*, the proportion of AT-rich DNA was 5.0%.

Using the suite of programs in PiRATE-Galaxy we predicted and classified transposable elements and SSRs from *B. fabae*. The same analysis was performed on the published *B. cinerea* B05.10 genome (van Kan et al., 2017) and the summary data are shown in Supplementary Table S2A. Although *B. cinerea* TEs have been identified and analyzed previously (Porquier et al., 2016), we applied the same identification methods for TEs from all three genome assemblies to enable direct comparison with the *B. fabae* assemblies. Overall, the numbers and sizes of transposable element classes for *B. fabae* were similar to *B. cinerea* B05.10. The most abundant previously identified TE classes for *B. cinerea* B05.10 were LTR and TIR and our analysis produced a similar LTR content but overestimated TIR and underestimated MITE (Porquier et al., 2016). Custom search tools SINE-Finder and MITE-Hunter (Supplementary Table S2B) for the targeted identification of SINEs and MITEs, respectively, came to similar conclusions as more generic repetitive element search tools. The amount of repetitive and transposable element sequence in *B. fabae* and *B. cinerea* was substantial, accounting for a total 1.8 Mb (4.2% of the genome) for *B. cinerea* and provisionally, 1.5–1.7 Mb (3.3 – 4.0% of the genome) for *B. fabae*. The data for *B. fabae* should be viewed with some caution because the identification of transposable elements from the more fragmented Illumina assemblies can be subject to errors due to mis-assembly of repetitive DNA sequence and misidentification.

From the respective lists of *B. fabae* proteins, we ran prediction pipelines for identifying Carbohydrate-Active enzymes (CAZymes) and putative effectors. Approximately 590 CAZymes were identified for *B. fabae* and this was fewer than the 681 predicted for *B. cinerea* using the same prediction method. The more complete genome assembly and more accurate gene model prediction for *B. cinerea* is likely to contribute to the greater number of CAZyme genes in *B. cinerea*, although there may also be genuine differences between species in the numbers of CAZyme genes. A summary of the numbers of predicted proteins in each CAZy class for *B. cinerea* and the two *B. fabae* isolates is given in the Supplementary Table S1. The number of proteins classified in each CAZy family within classes was highly correlated between *B. cinerea* and *B. fabae* (results not shown) and no clear differences in the complement of CAZymes between *Botrytis* species were found.

For the prediction of effector genes, we selected proteins based on the presence of a secretion signal peptide, small size, the number of cysteine residues and EffectorP version 2.0 score. We ran the effector prediction pipeline using several levels of stringency (Table 2) in which EffectorP score threshold was 0.8 or 0.6, mature protein MW cut-off was 25 to 35 kDa and minimum number of cysteines was 2 or 0. Table 3 shows the details of putative effectors predicted at the most stringent selection criteria of EffectorP score greater than 0.8 and MW less than 25 kDa. The putative effector genes were given provisional names where PE denotes “putative effector.” The predicted putative effectors in Table 3 were mostly described as hypothetical proteins, although *Bf*_PE-05, *Bf*_PE-07, and *Bf*_PE-27 were described as an endosomal cargo receptor, pectate lyase and long chronological lifespan protein, respectively. *Bf*_PE-06 and *Bf*_PE-28 had no BLAST hits and therefore have no functional annotations. *Bf*_PE-23 and *Bf*_PE-26 are necrosis and ethylene-inducing peptide-like proteins (NLP). The two paralogous NLPs in *B. fabae* have 86% amino acid sequence identity to one another and these two genes are direct orthologs of the *B. cinerea* NLP described as NEP1.

**TABLE 2:** Summary of effector prediction based on the protein annotations and tBLASTn from the two *Botrytis fabae* short read sequencing assemblies (*Bf*611 and *Bf*612) and two near continuous chromosome assemblies of *B. cinerea* isolate B05.10 and *Sclerotinia sclerotiorum* isolate 1980 using different selection criteria.

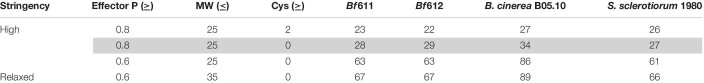

*All proteins have a secretion signal and effector predictions for the four genome annotations are based on EffectorP (v 2.0) scores, the molecular weight (MW) of the mature protein and the number of cysteines (Cys). Shaded in gray are the selection criteria for effector candidates that were analyzed in further detail (Table 3).*

**TABLE 3:** Predicted effectors for *B. fabae* using stringent conditions (EffectorP 2.0 ≥ 0.8, MW ≤ 25).

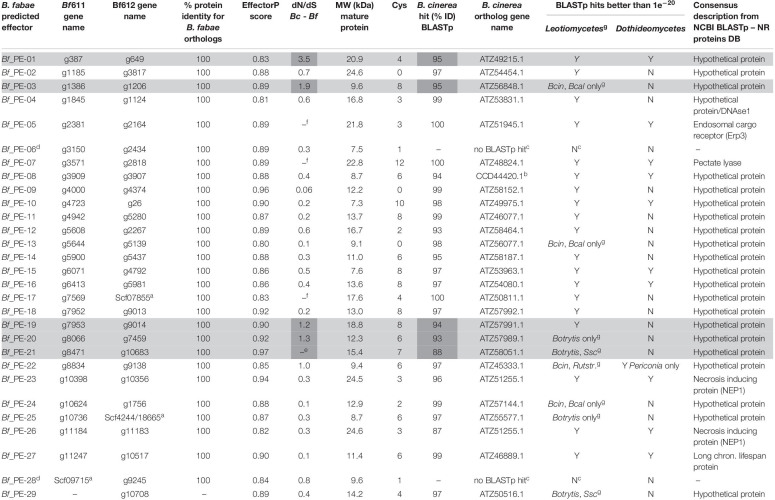

*The number of cysteine residues was not a criterion for selection but is included as a protein feature in the table. MW was calculated for the mature polypeptide after removal of the secretion signal predicted by SignalP 4.0. Putative function was inferred from the consensus of proteins with functional descriptions in BLASTp results. ^*a*^Orthologous proteins not detected by BLASTp of protein database due to mis-annotation or BLAST hit spanning two contigs. Evidence for ortholog gene was determined using tBLASTn of whole genome assembly and contig number of respective assembly is noted in the table. ^*b*^No BLASTp hit to annotated proteins for B05.10 but BLASTp hit to GenBank NR proteins. ^*c*^No BLASTp hit but tBLASTn hit to *B. cinerea* genome sequence in NR database. ^*d*^Putative orthologous pseudogenes in *B. cinerea*: single base mutation in *B. cinerea* PE-06 to a stop codon at amino acid position 41 out of 94. Five bp insertion in *B. cinerea* PE-28 pseudogene at bases 194-198 of 335 bp, with consequent frameshift. ^*e*^dN/dS Ratio was not calculated for PE-21 because dS was zero, there being 10 non-synonymous substitutions and 0 synonymous substitutions. ^*f*^Amino acid sequences from *B. fabae* and *B. cinerea* were identical. ^*g*^*Bcin, B. cinerea; Bcal*, *B. calthae*; *Ssc*, *S. sclerotiorum*; *Rutstr*, *Rutstroemia spp.**

**FIGURE 6:**
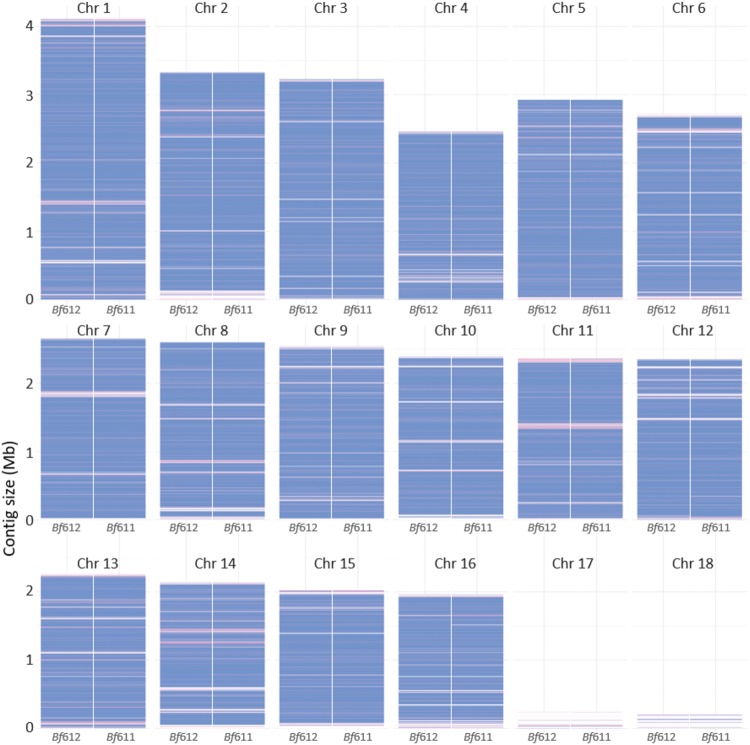
Alignment of contigs from the *Bf*611 and *Bf*612 *de novo* assemblies with the published *Botrytis cinerea* B05.10 reference genome sequence using NUCmer (Delcher et al., 1999; Kurtz et al., 2004). Contigs colored blue represent unique matches and red contigs represent non-unique matches to the *B. cinerea* B05.10 reference.

We aligned the *B. fabae* contigs to the published *B. cinerea* B05.10 chromosomal contigs using the NUCmer software and produced the graphical representation of coverage of the *B. fabae* genomes shown in Figure 6. *B. fabae* contigs are colored blue for unique matches to homologous sequence at the respective locations along the 18 *B. cinerea* chromosomal contigs. *B. fabae* contigs colored red are located at positions of non-unique matches to the *B. cinerea* contigs and these are likely to indicate regions of the *Botrytis* assemblies where there is repetitive or low-complexity sequence. Gaps between blue and red bars representing the *B. fabae* sequence were either not assembled or not sequenced correctly, or are deleted regions in the *B. fabae* genomes. Viewing the NUCmer comparison figure closely facilitates the identification of modest size gaps, and side-by-side comparison of two *B. fabae* isolates improves the confidence by which we can predict such gaps. It is worth noting that the smallest *B. cinerea* chromosomes, Chr 17 and Chr 18 were poorly matched to the *B. fabae* assemblies and it is likely that these small and possibly accessory chromosomes (van Kan et al., 2017) are absent from *B. fabae* as they are from *S. sclerotiorum* (Derbyshire et al., 2017). NUCmer analysis also reveals that non-unique matches and gaps were more prevalent at the ends of the homology-based putative chromosomes and this could be due to loss of sequence at chromosome ends in the actual *B. fabae* genomes or a greater abundance of repetitive DNA at the chromosome ends.

## Discussion

Here we have characterized the morphological and pathological features of two *B. fabae* isolates from chocolate spot lesions of faba beans. Furthermore, we present genome assemblies for *B. fabae* and use genome sequence comparison to identify differences in genome organization and gene complement with *B. cinerea*. We paid particular attention to predicted protein effector genes that might account for differences in host specificity and pathogenic lifestyle. With the potential for both *B. cinerea* and *B. fabae* to be isolated from diseased lentil crops we sought to document the growth characteristics of the isolates, *Bf*611 and *Bf*612 as verified examples of *B. fabae*. Assessment of growth rate and plate morphology for *B. fabae* compared with *B. cinerea* showed that the species have similar growth rate on solid media and only subtle differences in growth morphology. At 12 days after inoculation on 1/2 PDA plates, the difference in the degree of conidiation for *B. cinerea* compared to *B. fabae* was only marginally evident. With the addition of faba bean extract to growth media the increased production of conidia by *B. cinerea* was a clear distinguishing feature between the species and could be used for species differentiation. In addition, the production of sclerotia on faba bean extract-supplemented media for *B. fabae*, although variable between the two isolates, is another morphological feature that was distinct from *B. cinerea*. Production of spores on faba bean leaves overlaid on mycelial cultures of *B. fabae* isolates was similarly reduced in comparison to *B. cinerea* although sufficient spores for plant inoculations could be produced in this fashion. The difficulty in producing spores from *B. fabae* in culture has been noted in early studies (Last and Hamley, 1956; Leach and Moore, 1966; Harrison, 1983b; Harrison and Heilbronn, 1988) and a growth medium supplemented with 10% (v/v) sucrose, Medium X, has been used to promote sporulation in culture (Last and Hamley, 1956). We tested Medium X in this study but we were unsuccessful in producing *B. fabae* conidia using this growth media and therefore, we developed the simple host leaf overlay method for spore production. Spore sizes for *B. fabae* and *B. cinerea* were as reported previously (Harrison, 1983a; Bayaa and Erskine, 1998; Lindbeck et al., 2009) and provide a reliable method for differentiating the two species. The conformation of conidiophores revealed by light microscopy for the two species reflects their respective conidial size, with those of *B. cinerea* being more compact. The cultural and morphological investigations in this study were in agreement with previous studies and we confidently align our published genome assembly data with the description and characterization of *B. fabae* presented here. It is important to stress that comparisons made between *B. fabae* isolates from faba bean may differ from those found on lentils. Similarly, our chosen *B. cinerea* control strain Bc7 may differ from B05.10, thereby potentially confounding our conclusions in a genomic sense. Bc7 may differ from *B. cinerea* isolates from BGM infections of lentils and this may have implications for our pathology studies.

In a comprehensive review of screening techniques for necrotrophic fungal diseases of legumes, screening of *B. fabae* infections has been reported to be performed in field inoculation of faba bean plants two months after sowing with 4–5 × 10^5^ spores.mL^–1^ or by application of 5 × 10^5^ spores.mL^–1^ to detached faba bean leaves (Tivoli et al., 2006). Other researchers have similarly used field and detached-leaf screening for measuring disease responses of faba bean germplasm to *B. fabae* (Hanounik and Robertson, 1988; Bouhassan et al., 2004; Villegas-Fernández et al., 2012). Our *B. fabae* seedling assays revealed similar levels of aggressiveness on the susceptible faba bean variety Ascot and the moderately susceptible variety PBA Rana, even with the lower inoculum concentration of 4 × 10^4^ spores.mL^–1^. In a third experiment, disease scores were reduced for Ascot, PBA Rana and PBA Samira compared with the earlier experiments, however, the more susceptible variety to chocolate spot was the most susceptible in the seedling assays and the MS-rated varieties PBA Rana and PBA Samira were more resistant. Although *B. cinerea* could infect faba beans, lesions were small and did not develop to large-sized lesions over time. Our results reaffirm that *B. cinerea* is not likely a significant contributor to chocolate spot disease in faba beans. Early studies were not always in agreement about the role of *B. cinerea* in contributing to chocolate spot in faba bean with both species commonly being isolated from diseased faba bean plants (Harrison, 1983a, 1988). Other studies have demonstrated that *B. fabae* is more aggressive toward faba bean, and lesion development after inoculation with *B. cinerea* is restricted or inhibited (Mansfield and Deverall, 1974b). For lentil, the relative contribution of *B. fabae* and *B. cinerea* to BGM is less clear, with reports of the major causal *Botrytis* pathogen in some lentil-growing regions being *B. cinerea* (Bayaa and Erskine, 1998; Tivoli et al., 2006). In Australia, both species can be found on BGM-infected lentil (Lindbeck et al., 2008), and Davidson and Krysinska-Kaczmarek (2007) demonstrated that both species could infect and produce BGM symptoms under controlled conditions (Davidson and Krysinska-Kaczmarek, 2007). For lentil production in Canada, *B. fabae* is suspected to be more important than *B. cinerea* (Davidson and Krysinska-Kaczmarek, 2007). Our seedling assay results for three lentil cultivars suggest that *B. fabae* is an important agent in BGM epidemics of lentil, with significantly higher disease scores for the two *B. fabae* isolates on lentil cultivars compared to *B. cinerea*. Of note was that the Kaspa field pea control was completely resistant to both *Botrytis* species. Clearly, *B. fabae* is a key pathogen for faba beans and lentils and further research is required to determine relative proportions of respective species in natural disease epidemics in crop production situations. While in the past, pathologists have relied on species identification by spore size morphology, variation in spore production capacity would likely bias estimations of respective species in infected crops. More up to date and specific methods for species quantitation based on fungal genomic DNA sequence should be implemented to more accurately assess the contribution of respective species to lentil BGM. More detailed knowledge of the relative importance of the different *Botrytis* species to crop diseases will enable more targeted plant breeding efforts that deal with the key causal species and the specific host responses to the respective pathogens.

Published comparisons of the host responses of faba bean to *B. cinerea* and *B. fabae* describe localized dark brown discoloration or necrosis at the site of infection (Mansfield and Deverall, 1974b) for *B. cinerea* and spreading necrotic lesions for *B. fabae*. Using light microscopy we observed different reactions in faba bean for *B. cinerea* and *B. fabae* infection. *B. cinerea* inoculation produced reddish-brown coloration in epidermal cells at the site of presumed penetration or perception of hyphae on the leaf surface. Further growth of hyphae was limited or perhaps completely inhibited in what appears to be a specific resistance response to *B. cinerea*. In contrast, symptoms in faba bean after *B. fabae* inoculation was characterized by spreading brown colored necrosis and proliferation of mycelial growth. The dissimilar reactions in faba bean leaves to the two *Botrytis* species exemplifies the distinct incompatible and compatible responses that are possibly determined by specific host immunity mechanisms toward *B. cinerea*. These might be a general pathogen-associated molecular pattern or PAMP-triggered immune (PTI) response or more specific, effector-triggered immune (ETI) responses. Faba beans are known to produce the phytoalexin wyerone acid and the production of this compound may be associated with the reddish brown coloration of host cells at the sites of interaction with the pathogen. *B. cinerea* is more sensitive to wyerone acid and fungal growth can be inhibited at low concentration of the compound (Mansfield and Deverall, 1974a). *B. fabae* is more tolerant of wyerone acid and can metabolize the phytoalexin to less toxic by-products to overcome host resistance and a compatible pathogen-host interaction is the likely consequence of this toxin catabolism (Mansfield and Deverall, 1974a; Harrison, 1988). Effector proteins are likely to be synthesized by *B. fabae* to induce effector-triggered susceptibility in faba bean, thereby leading to the compatible disease response and host susceptibility. The generalized necrosis reaction that we observed in lentil resembles the compatibility in faba bean and points to a similar level of susceptibility and likely virulence mechanism in *B. fabae* toward lentil. In addition, the more resistant lentil variety PBA Jumbo2 exhibited reduced necrosis but a similar level of mycelial growth to the more susceptible variety PBA Bolt. We suspect that *B. fabae* is not actively suppressed by the host in either lentil or faba bean as seems to be the case for *B. cinerea* in faba bean.

To date no whole genome assembly for *B. fabae* has been described, despite extensive genome sequencing and analysis for the more prominent, generalist leotimycete species, *B. cinerea* (Amselem et al., 2011; van Kan et al., 2017) and *S. sclerotiorum* (Amselem et al., 2011; Derbyshire et al., 2017). In addition to these reference genomes for model species, nine further *Botrytis* species have been sequenced and a pan-genome for *Botrytis* has been constructed (Valero-Jiménez et al., 2019). The genome sequencing and assembly of two *B. fabae* isolates described here is a major advance for the study of chocolate spot and BGM in faba beans and lentils. The availability of a whole genome sequence assembly for *B. fabae* will facilitate development of DNA metabarcoding and quantitative RT-PCR methods for accurate quantitation of inoculum of different *Botrytis* species in disease epidemics, seed, soils and crop residues. It should be noted that there are limitations in genome assembly from Illumina sequencing data and these have consequences for gene and transposable element annotation. Although highly fragmented, these *B. fabae* genome assemblies are useful for the identification of putative effector and secondary metabolite genes. However, caution should be exercised in making definite conclusions about the species from this study.

Our genome sequencing of two *B. fabae* isolates by Illumina sequencing produced assembly sizes of approximately 44 Mbp, which was similar to that of other *Botrytis* species. *B. cinerea* has genome size of 43.5 Mbp (van Kan et al., 2017) and for nine recently published genome assemblies for Botrytis species, genome sizes range from 43 to 55 Mbp (Valero-Jiménez et al., 2019). Sequencing statistics presented here were within a similar range for other *Botrytis* Illumina sequencing projects with similar magnitude for N50, largest contig, sequencing coverage and total number of contigs (Amselem et al., 2011; Valero-Jiménez et al., 2019). Whole proteome ortholog group membership for our analysis using OrthoFinder found proteins in *B. fabae* not present in *B. cinerea*, similar to the finding for secreted proteins from nine *Botrytis* species with narrow host specificity, in contrast to the generalist model organism, *B. cinerea* (Valero-Jiménez et al., 2019). The number of protein orthogroups that distinguish the two Botrytis species seems quite high with more than 2,000 orthogroups unique to either *B. cinerea* or *B. fabae*. tBLASTn of all *B. fabae* predicted proteins against the B05.10 genome assembly found only 77 proteins for *Bf*611 and 135 proteins for *Bf*612 for which corresponding DNA sequence was not found in B05.10. Many of these had no homologous nucleotide sequence in B05.10 or were putative pseudogenes. However, annotated orthologous proteins for these proteins could be found in other fungi using BLASTp. Future improvements in genome assemblies through long-read sequencing and improved annotations from *B. fabae* RNAseq data will lead to better quality genomes for the species.

Alignment of *B. fabae* contigs with the *B. cinerea* PacBio assembly revealed a high level of similarity with most genomic regions of the 18 chromosomal contigs for *B. cinerea* B05.10. Notwithstanding this level of sequence similarity, there were sections of the B05.10 genome that were not represented in the *B. fabae* contigs. Moreover, discrete sections of non-unique or repetitive DNA were evident by alignment of *B. fabae* contigs to the reference *B. cinerea* assembly. Further analysis of repetitive sequence was achieved using the OcculterCut program (Testa et al., 2016) by which we identified a unique feature of the genome architecture of *B. fabae*. *B. fabae* has approximately 10–12% of the genomic sequence with highly AT-rich DNA whereas by our estimation, *B. cinerea* B05.10 has only 5% AT-rich DNA sequence. Published estimation of AT-rich DNA content of *B. cinerea* isolate T4 is 4.6% and other estimates for AT-rich DNA content include *S. sclerotiorum* 1980 (0%) and *Leptosphaeria maculans* v23.12.3 (36.7%) (Testa et al., 2016). Biotrophic mildew and rust species, *Blumeria graminis* f. sp. *tritici* and *Puccinia graminis* f. sp. *tritici* have a single peak of GC-content centered around 40% and have no AT-rich regions (Testa et al., 2016). The presence of a greater proportion of AT-rich DNA in certain fungal genomes results from the combination of transposon insertion and activity, and the phenomenon of repeat-induced point mutation (RIP) where cytosine bases in repeat DNA regions are mutated to thymine bases (C to T transition). Consequently, repetitive DNA becomes more AT-rich over time (Hane and Oliver, 2008; Fudal et al., 2009; van de Wouw et al., 2010; Testa et al., 2016). AT-Rich DNA regions in plant pathogenic fungi are of particular interest due to them being indicative of transposon and RIP activity that drive evolution and adaptation of pathogen populations in the selective environment of plant host species and cultivars in which there is variation in disease resistance. Virulence genes such as those that encode effectors are often located near or within these repetitive, and potentially mobile regions of the genome (Raffaele and Kamoun, 2012; Faino et al., 2016). In addition, these genes may be subject to higher mutation frequencies due to RIP and thus, the distribution of GC-content and the presence of AT-rich DNA is a signature feature of pathogens that have evolved host specificity for a discrete host species or a small group of related hosts (Fudal et al., 2009; van de Wouw et al., 2010). The observed GC-content distribution and the significant proportion of AT-rich DNA is consistent with the hypothesis that *B. fabae* may have evolved specific mechanisms to overcome the host defenses of faba bean and lentil. Low disease scores for faba bean and lentil infection, and microscopic evidence for a specific, localized immune response in faba bean to *B. cinerea* supports the notion that *B. fabae* has evolved genetic mechanisms for pathogenicity that are distinct from *B. cinerea*.

We investigated the transposable elements and repetitive DNA sequences using the PiRATE-Galaxy pipeline and we found small differences in some classes of repetitive and transposable elements between species. The total nucleotide count for all repetitive and TE DNA was similar for *B. cinerea* and *B. fabae*, accounting for around 3–4% of the genome. Minor variations in proportions of TE classes between *B. cinerea* and *B. fabae* are consistent with different transposable element activity among these species. However, a more detailed study with a greater number of isolates would be required to compare any effects of transposable elements on the evolution of host specificity in *Botrytis*. At this stage, the reasons for the differences in% GC content between *B. cinerea* and *B. fabae* remain unresolved.

Effector prediction from microbial pathogen genomes is based on the premise that effector proteins are secreted to the site of action in the plant-pathogen interaction and therefore carry a signal peptide. Furthermore, effector proteins are generally of small size and often carry a proportionally high number of cysteine residues to counter the highly reducing environment of the plant apoplast (Jones et al., 2018). Effector proteins are subject to constant evolution of primary structure while maintaining secondary and tertiary structural elements in order to modify surface chemical properties and topography. Effectors evolve either to evade recognition by the host in the case of Avr proteins, or to adapt to host receptor evolution and to maintain virulence in the case of necrotrophic effectors. Thus, effector gene sequences that confer host-specificity co-evolve with the host in a pathosystem, and divergence of effector sequences across evolutionary history and speciation among related genera lead to low levels of sequence conservation between effectors from pathogenic fungal species (Sánchez-Vallet et al., 2018). We ran our effector prediction bioinformatics pipeline at different levels of stringency and at each level we identified fewer predicted effector proteins for *B. fabae* than for *B. cinerea* B05.10 (van Kan et al., 2017). These differences are likely due to the more complete assemblies and more accurate annotation using RNAseq for *B. cinerea*. Nevertheless, we identified 29 putative effectors from the *B. fabae* genome assemblies. One of these was predicted for *Bf*612 but not found in *Bf*611. The dN/dS ratio is a measure of positive or diversifying selection between orthologous proteins from different species. This is where a gene has accumulated and retained mutations that generate amino acid polymorphisms likely to confer adaptive changes in protein function. Such changes are of particular importance to small interacting proteins such as effectors, where the function is for recognition by host receptors in effector-triggered susceptibility, or for evasion of recognition in the case of effector-triggered immunity or avirulence. Table 3 shows the top 29 *B. fabae* predicted effectors with their protein properties and a summary of BLAST results. Average mature protein size was 14.4 kDa and the smallest proteins were <8 kDa. Except in the case of one protein (PE-29), where orthologs were not detected between *B. fabae* isolates, predicted effector amino acid sequences were identical between isolates. Absence of particular *bona fide* effectors between isolates could theoretically determine variation in host cultivar specificity of different isolates within a species. With the current assemblies for *B. fabae* constructed from short-read Illumina data, we cannot rule out that this case of gene absence was not due to mis-assembly. BLAST searches of better-characterized genomes using non-redundant protein or nucleotide databases revealed that most of the predicted effectors from *B. fabae* were hypothetical proteins with no matches to characterized proteins with known functions. Two proteins (PE-06 and PE-28) had no hits to any database sequences and there were two predicted NEP or NPP-type proteins (PE-23 and PE-26). The necrosis and ethylene-inducing peptide-like protein effectors (NEP) are non-host-specific effectors that are widespread among microbial plant pathogens (Gijzen and Nurnberger, 2006; Staats et al., 2007). Interestingly, the two NEP (NLP) paralogs in *B. cinerea* share only 39% identical amino acid sequence, whereas PE-23 and PE-26 are more highly conserved, with 86% sequence identity. We speculate that the *B. fabae* NLP effectors are orthologous proteins of *B. cinerea* NEP1 rather than each being an ortholog of the respective NEP1 and NEP2 proteins that are more highly diverged in *B. cinerea*. We found no ortholog for the *B. cinerea* NEP2 in *B. fabae* and the two NLPs in *B. fabae* either have originated from a single ancestral gene orthologous to *B. cinerea* NEP1 or the divergence and evolution of the two NLP genes has occurred at different rates in the two species.

*Botrytis fabae* genes for PE-04, PE-06, and PE-27 have functional descriptions for homologs in the NR database and were not considered to be likely effectors. The best candidates for genuine effector function are those with few BLAST hits in closely related leotiomycete species and preferably no hits in the *dothideomycetes*. There were 18 proteins among the 29 most likely effector candidates with no known function and no BLAST hit in the *dothideomycetes*. Most of these had a dN/dS ratio of less than 1.0, indicating purifying selection during evolution, and therefore conservation of sequence or low level of divergence through evolution in accord with the natural rate of mutation. Five predicted *B. fabae* proteins, PE-01, PE-03, PE-19, PE-20, and PE-21 have no putative function based on BLAST results but have dN/dS ratios that suggest positive, or diversifying selection with dN/dS ratios above 1.0 and as high as 3.5. The most extreme case was for PE-21 where all ten nucleotide substitutions were non-synonymous and there were no synonymous substitutions. PE-03 is a homolog of BC1G_11117 (ATZ56848) that has been characterized as a hydrophobin homolog in *B. cinerea* (Mosbach et al., 2011). PE-18 and PE19 are adjacently located on a single *B. fabae* contig and the homologous region in *B. cinerea* B05.10 was 85 Kb distant from a large 50 Kb repetitive section of DNA. The B05.10 PE-20 ortholog was also found at this location, approximately 3 Kb from the PE-19 ortholog. We explored this region further in B05.10 and found another putative effector (signal peptide, mature MW 6.7 KDa, EffectorP 2.0 score 0.7) with *B. fabae* orthologs and located 12.5 Kb from the PE-19 ortholog. Also in the region and 27 Kb from the nearby AT-rich region was the PE-21 ortholog, suggesting that this region of *B. cinerea* chromosome 15 and homologous *B. fabae* chromosomes could be a hotspot for effectors.

Direct prediction of effector proteins is not an exact science and further studies such as transcriptome analysis and functional characterization of candidate genes for validation as genuine effectors is required. This list provides a starting point for future investigations of the role of protein effectors in the virulence of *B. fabae*.

Secondary metabolite clusters in pathogenic fungi are often responsible for the synthesis of virulence factors, toxins or effectors. In *B. cinerea*, examples of phytotoxic metabolites include botrydial and botcinic acid (Collado and Viaud, 2016). In the recent comparison of *Botrytis* genomes (Valero-Jiménez et al., 2019), homology searches for known *B. cinerea* secondary metabolite clusters from nine *Botrytis* species revealed variation in the complement of secondary metabolite synthesis genes. We applied a homology search similar to that of Valero-Jiménez et al. (2019) for the two *B. fabae* genomes using the same 43 genes from *B. cinerea* that represent each of the proposed or verified secondary metabolite gene clusters. We also ran antiSMASH on the *B. fabae* assemblies but found no additional secondary metabolite clusters other than those already identified through BLAST searches with the *Botrytis* query set of 43 protein sequences. Genes for seven key enzymes were absent from *B. fabae*. In terms of the complement of secondary metabolite genes, *B. fabae* was most similar to *B. cinerea* and *B. calthae*, and the presence/absence of secondary metabolite genes for other *Botrytis* species and *S. sclerotiorum* approximately matched the phylogeny based on or single-copy ortholog genes described by Valero-Jiménez et al. (2019). *B. cinerea* produces two secondary metabolite phytotoxins, namely botrydial and botcinic acid (Dalmais et al., 2011; Collado and Viaud, 2016; Porquier et al., 2016). Botcinic acid is produced by enzymes from two proximate gene clusters characterized by key enzymes BcPKS6 (syn BcBOA6) and BcPKS9 (syn BcBOA9). The two closely located clusters contain 13 genes, named BOA1 to BOA13 in positional order along the respective clusters. In *B. fabae*, the two key PKS genes are present with almost 100% nucleotide identity to the *B. cinerea* orthologs, as are all other genes from the two clusters (Dalmais et al., 2011; van Kan et al., 2017; Porquier et al., 2019). A significant finding from the genome sequencing of *B. fabae* is that six of the seven genes from the botrydial gene cluster were not present in the genome assembly. Only the P450 monooxygenase BcBOT4 ortholog is present in the *B. fabae* genome assembly as a remnant gene from the cluster. We confirmed the loss of the region using BLASTn and tBLASTn searches (Altschul et al., 1990) of the *B. fabae* assemblies and also the raw reads for both sequencing projects. The botridial gene cluster in *B. cinerea* is partitioned by two AT-rich regions of approximately 8 Kb and 10 Kb, which have several transposable elements (Porquier et al., 2016). A smaller 1 Kb AT-rich sequence is located between BcBOT4 and four other BcBOT genes (BcBOT1, 2, 3, and 5) and there is a repetitive 1 Kb sequence with a transposable element adjacent to BcBOT7 at the end of the cluster (Porquier et al., 2016). It is plausible that these small repetitive DNA sequences have contributed to the loss of much of the botrydial cluster in *B. fabae*. We suspect that the loss of this well characterized toxin has phenotypic, and possibly host specificity consequences for the species. For *B. cinerea*, loss of botrydial (Siewers et al., 2007) and botcinic acid (Dalmais et al., 2011) by gene deletion reduced the virulence of the pathogen when both metabolites were absent. It is possible that the loss of a secondary metabolite toxin could change the way that *B. fabae* is recognized in faba bean in comparison to *B. cinerea*, and the mode of infection and host interaction that it employs. In addition to these differences in secondary metabolite gene clusters, biosynthesis genes for abcisic acid (ABA) were missing in *B. fabae*, as were a further three PKS genes and one NRPS. ABA is produced by *B. cinerea* although a role other than in possible host manipulation has not been determined (Collado and Viaud, 2016). The four other putative gene clusters present in *B. cinerea* and missing in *B. fabae* produce hypothetical secondary metabolites with uncharacterized structures and biological functions (Collado and Viaud, 2016). Possible roles in virulence or host interaction of *B. cinerea* with susceptible plant species, and the possibility of beneficial consequences of gene loss for *B. fabae*, can not be ruled out.

The published genome assemblies that we present here will provide the basis for future investigations of *B. fabae* in causing chocolate spot and BGM in faba bean and lentil. Perhaps the most immediate advance that could be realized in the short term will be the use of genomic sequence to better detect and differentiate *Botrytis* species in legume crops. More long term developments in *B. fabae* research will be to use the published genome assemblies to investigate the molecular mechanisms of disease causation and virulence with the putative protein effector and secondary metabolite genes discussed here as a good starting point for further studies.

## Data Availability Statement

The datasets generated for this study can be found in the PRJNA505227, GCA_004335035.1, and GCA_004335055.1.

## Author Contributions

RL designed the experiments. LF-C and RL performed the isolate characterization and pathology work. LF-C and RS carried out the genome sequencing. RS and JD produced genome assemblies, annotation and analysis. LF-C, RS, JD, and RL analyzed the data. RL wrote the manuscript with input from LF-C, RS, and JD. All authors reviewed the manuscript.

## Conflict of Interest

The authors declare that the research was conducted in the absence of any commercial or financial relationships that could be construed as a potential conflict of interest.

## Footnotes

1 http://www.fao.org/faostat/en/#data

2 http://www.bioinformatics.babraham.ac.uk/projects/fastqc

3 http://www.repeatmasker.org/

4 https://www.ebi.ac.uk/about/vertebrate-genomics/software/exonerate

5 https://my.vmware.com/en/web/vmware/downloads

6 http://www.repeatmasker.org

7 https://github.com/gatechatl/dNdS-Calculator

8 https://github.com/ebi-pf-team/interproscan

9 https://www.ebi.ac.uk/interpro/interproscan.html

10 http://www.bom.gov.au/climate/data/

11 http://www.pbseeds.com.au/docs/PBA-Jumbo2-2014.pdf

## Supplementary Material

FIGURE S1 BUSCO assessment of genome completeness for *B. fabae Bf*611 and *Bf*612, and *B. cinerea* B05.10.

FIGURE S2 Orthofinder analysis of *Bf*611, *Bf*612, and *B. cinerea* B05.10.

TABLE S1 CAZyme classifications for predicted CAZymes of *Bf*611, *Bf*612, and *B. cinerea* B05.10.

TABLE S2 Transposable and repetitive elements for *Bf*611, *Bf*612, and *B. cinerea* B05.10.

DATA SHEET S1 FASTA *B. cinerea* putative effectors.

DATA SHEET S2 FASTA *Bf*611 proteins.

DATA SHEET S3 FASTA *Bf*611 putative effectors.

DATA SHEET S4 FASTA *Bf*612 proteins.

DATA SHEET S5 FASTA *Bf*612 putative effectors.
