# Supplementary material for: Characterization of Growth Morphology and Pathology, and Draft Genome Sequencing of Botrytis fabae, the Causal Organism of Chocolate Spot of Faba Bean (Vicia faba L.)
Source: Front Microbiol. 2020 Feb 18;11:217. doi: 10.3389/fmicb.2020.00217 (PMC7040437; doi:10.3389/fmicb.2020.00217)
Supplement: FIGURE S1 — BUSCO assessment of genome completeness for B. fabae Bf611 and Bf612, and B. cinerea B05.10. [file Data_Sheet_6.PDF]

Supplementary Figures:

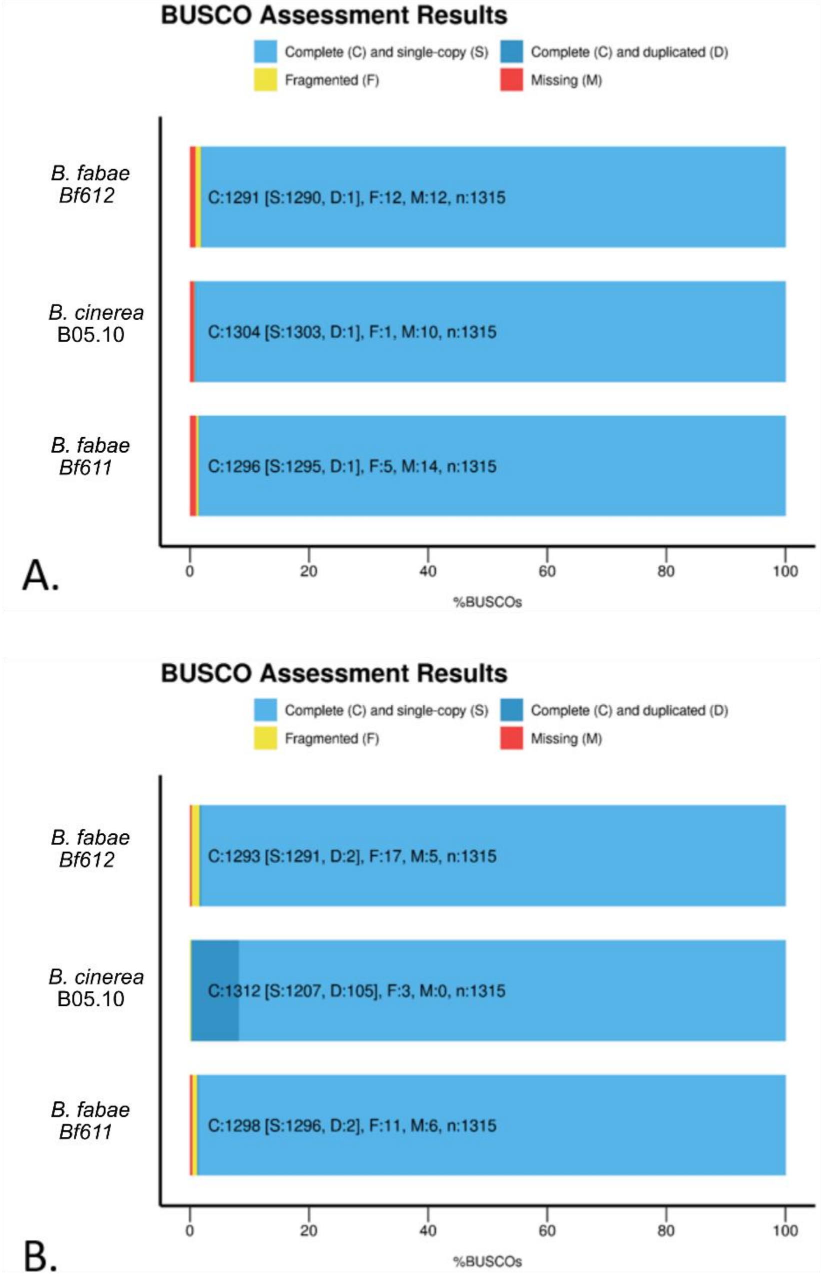

Figure S1. Analysis of genome completeness using BUSCO. Annotated *B. fabae* genome assemblies for isolates *Bf*611 and *Bf*612, and *B. cinerea* B05.10 (van Kan et al., 2017) were analysed using 1,315 universal single-copy ortholog genes from the BUSCO website https://busco.ezlab.org/. A., Genome analysis and B., proteome analysis.

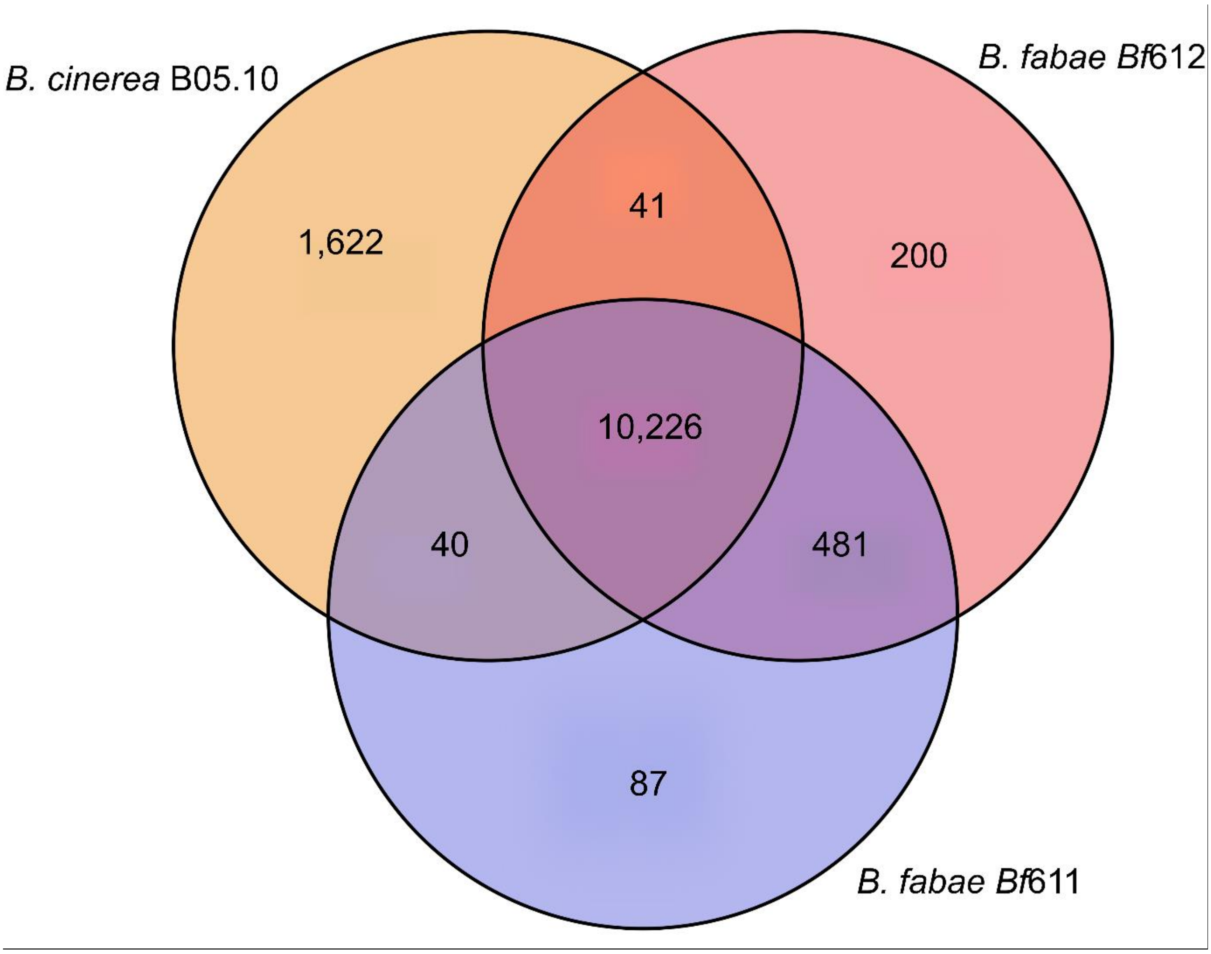

Figure S2. Venn diagram showing the numbers of annotated orthologous gene groups shared between *Botrytis* species and between isolates of *B. fabae* isolates, *Bf611* and *Bf*612, generated using the OrthoFinder software package.
