# Supplementary material for: Characterization of Growth Morphology and Pathology, and Draft Genome Sequencing of Botrytis fabae, the Causal Organism of Chocolate Spot of Faba Bean (Vicia faba L.)
Source: Front Microbiol. 2020 Feb 18;11:217. doi: 10.3389/fmicb.2020.00217 (PMC7040437; doi:10.3389/fmicb.2020.00217)
Supplement: TABLE S1 — CAZyme classifications for predicted CAZymes of Bf611, Bf612, and B. cinerea B05.10. [file Data_Sheet_7.PDF]

Supplementary Tables:

Table S1. Number of Carbohydrate-Active Enzymes by family predicted from *B. cinerea* B05.10, and *B. fabae Bf*611 and *Bf*612.

**CAZy family**	**Description**	**Bc B05.10**	***Bf*611**	***Bf*612**
AA	Multicopper oxidases	107	93	93
CBM	Carbohydrate-binding module	6	6	6
CE	Carbohydrate esterase	82	72	71
GH	Glycoside hydrolase	263	226	229
GT	Glycosyl transferase	88	75	74
PL	Polysaccharide lyase	10	13	11
N	Un-classified	125	104	104
	Total	681	589	588

Table S2. Number of sequences in genome assemblies classified as transposable elements or SSRs, and total and average size for each type. TE content is also presented as a percentage of the assembly size for each species / strain (in parentheses). Note: The *B. cinerea* B05.10 genome assembly was a complete reference assembly consisting of 18 full-length chromosomes and the *B. fabae* assemblies were more fragmented draft assemblies from Illumina data. (A) TEs and repetitive elements detected and classified using programs other than MITE-Hunter and SINE-Finder in the PiRATE suite of programs. (B) MITEs and SINEs detected using specific TE-detection programs in the PiRATE pipeline.

A.

**Class**	**Type**	**Number of sequences**			**Total nucleotides**			**Average size**		
		B05.10	Bf611	Bf612	B05.10	Bf611	Bf612	B05.10	Bf611	Bf612
**I**	LARD	3	4	4	18945 (0.04)	13139 (0.03)	18087 (0.04)	6315	3285	4522
	LINE	3	7	20	4793 (0.01)	8950 (0.02)	29737 (0.07)	1598	1279	1487
	SINE	-	7	2	-	2810 (0.01)	1037(0.002)		401	201
	LTR	335	679	637	993593 (2.3)	745362 (1.7)	835128 (1.9)	2966	1098	1311
	TRIM	11	-	2	14344 (0.03)	-	1120 (0.003)	1304	-	560
**II**	MITE	23	5	3	11030 (0.03)	1894 (0.004)	999 (0.002)	480	379	333
	TIR	144	341	351	204319 (0.5)	311430 (0.7)	360917 (0.8)	1419	913	1028
	Helitron	-	1	1	-	1920 (0.004)	1920 (0.004)	-	1920	1920
**SSR**	SSR	238	271	246	366321 (0.8)	231941 (0.5)	314515 (0.7)	1539	856	1279
	No Cat	226	297	253	210334 (0.5)	137284 (0.3)	133247 (0.3)	931	462	527
	Host gene	4	1	4	14404 (0.03)	10912 (0.02)	31034 (0.07)	3601	10912	7758

B.

**Program**	**Number of sequences**			**Total nucleotides**			**Average size**		
	B05.10	Bf611	Bf612	B05.10	Bf611	Bf612	B05.10	Bf611	Bf612
**MITE-Hunter**	12	7	14	4259	1678	2774	355	240	251
**SINE-Finder**	21	13	17	6388	4425	5307	319	340	312
